# Surgical bailout for underexpanded transcatheter mitral valve replacement: addressing hemodynamic challenges

**DOI:** 10.1093/ehjcr/ytaf143

**Published:** 2025-03-24

**Authors:** Chadi Aludaat, Arnaud Gay, Quentin Landolff

**Affiliations:** Department of Thoracic and Cardivascular Surgery, Rouen University Hospital, CHU de Rouen, 1 Rue de Germont, Rouen 76000, France; Department of Cardiology, Clinique du Cèdre, 950 rue de la Haie, Bois Guillaume 76230, France; Department of Cardiology, Clinique Saint-Hilaire, 2 place Saint-Hilaire, Rouen 76000, France

An 81-year-old man underwent transseptal transcatheter mitral valve-in-valve replacement (*Panels A* and *B*) using a 29 mm Sapien 3 (Edwards Lifesciences) for a failing 31 mm Mosaic bioprosthesis (Medtronic). Initially, the procedure yielded favourable haemodynamic and clinical outcomes. However, 6 months later, the patient presented with dyspnoea and an elevated mean transprosthetic gradient (11 mm Hg).

**Figure:**
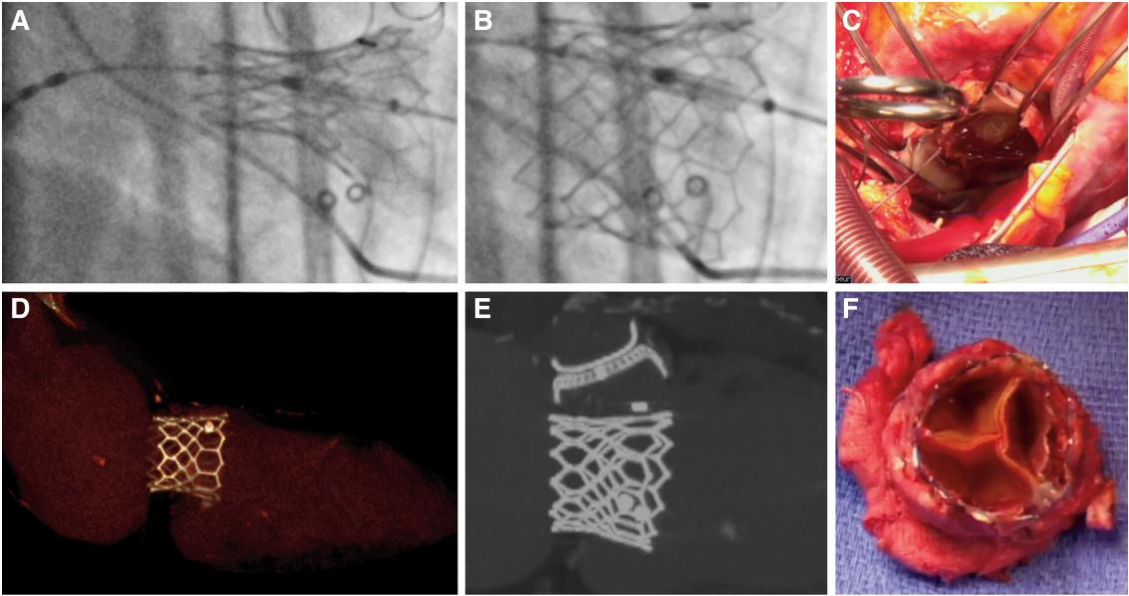


A comprehensive evaluation excluded infective endocarditis. Echocardiography confirmed prosthesis–patient mismatch, while gated cardiac computed tomography ruled out leaflet thrombosis or hypoattenuated leaflet thickening. Instead, incomplete valve expansion, which is common in valve-in-valve procedures, was identified as the cause of the elevated gradients. This phenomenon arises when the surgical valve ring constrains the transcatheter heart valve (THV), resulting in suboptimal expansion (*Panels D* and *E*), reduced effective orifice area, and impaired haemodynamics. Although this issue is well recognized in aortic valve-in-valve cases, similar findings are less documented in mitral transcatheter mitral valve replacement (TMVR).

Given these findings, the multidisciplinary cardiac team determined that post-dilatation was not warranted, as the initial balloon inflation was performed with an additional 2 cc volume. Surgical reintervention was performed despite the elevated risk, resulting in a successful bioprosthetic valve replacement. Intraoperative examinations (*Panels C* and *F*) confirmed the absence of structural damage or thrombotic complications. Postoperatively, the mitral mean gradient normalized (6 mm Hg), and the patient exhibited significant clinical improvement.

This case highlights incomplete THV expansion as a cause of residual gradient in TMVR, emphasizing the importance of preprocedural planning and imaging. Although TMVR is a less invasive option for failed surgical prostheses, individualized approaches remain essential. As this field rapidly evolves, further advancements in THV design and deployment techniques are critical for minimizing complications and optimizing outcomes.

Multimodal imaging and surgical findings of valve-in-valve transcatheter mitral valve replacement using the Edwards Sapien 3 transcatheter heart valve. (*A*) Fluoroscopic view during deployment of the Sapien 3 transcatheter heart valve in a failed mitral bioprosthesis. (*B*) Post-deployment fluoroscopic view showing optimal positioning of the Sapien 3 transcatheter heart valve within the bioprosthesis. (*C*) Intraoperative view through the left atrium, demonstrating valve-in-valve implantation. (*D*) Gated computed tomography scan with 3D reconstruction highlighting the Sapien 3 transcatheter heart valve constrained by the pre-existing bioprosthesis, preventing complete circular expansion of the stent frame. (*E*) Coronal computed tomography scan showing the constrained Sapien 3 transcatheter heart valve stent frame within the bioprosthesis. (*F*) Macroscopic view of the explanted Sapien 3 transcatheter heart valve demonstrating stent-frame deformation inside the bioprosthesis.

## Data Availability

The data underlying this article will be shared upon reasonable request by the corresponding author.

